# Nanoclays in medicine: a new frontier of an ancient medical practice

**DOI:** 10.1039/d2ma00528j

**Published:** 2022-08-31

**Authors:** Kalpana S. Katti, Haneesh Jasuja, Sharad V. Jaswandkar, Sibanwita Mohanty, Dinesh R. Katti

**Affiliations:** Department of Civil Construction and Environmental Engineering, North Dakota State University Fargo ND 58105 USA Kalpana.Katti@ndsu.edu 701-231-9504

## Abstract

Clays have been used as early as 2500 BC in human civilization for medicinal purposes. The ease of availability, biocompatibility, and versatility of these unique charged 2D structures abundantly available in nature have enabled the extensive applications of clays in human history. Recent advances in the use of clays in nanostructures and as components of polymer clay nanocomposites have exponentially expanded the use of clays in medicine. This review covers the details of structures and biomedical applications of several common clays, including montmorillonite, LAPONITE®, kaolinite, and halloysite. Here we describe the applications of these clays in wound dressings as hemostatic agents in drug delivery of drugs for cancer and other diseases and tissue engineering. Also reviewed are recent experimental and modeling studies that elucidate the impact of clay structures on cellular processes and cell adhesion processes. Various mechanisms of clay-mediated bioactivity, including protein localization, modulation of cell adhesion, biomineralization, and the potential of clay nanoparticles to impact cell differentiation, are presented. We also review the current developments in understanding the impact of clays on cellular responses. This review also elucidates new emerging areas of use of nanoclays in osteogenesis and the development of *in vitro* models of bone metastasis of cancer.

## Abstract 2

Clays have been used as early as 2500 BC in human civilization for medicinal purposes.

## Graphical abstract

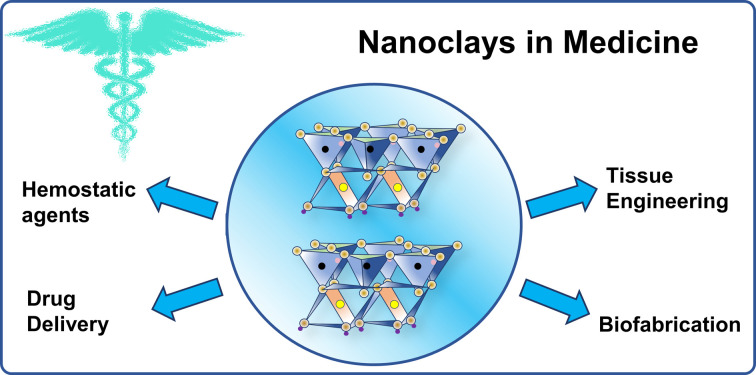

## 1. Introduction

Designing advanced biomaterials with controlled physical, chemical, electrical, and biological properties, to facilitate the formation of functional tissues holds enormous promise in biomedical applications.^1^ Clay minerals are an emerging class of biomaterials owing to their thickness that enables nanoscale characteristics, charged and biocompatible surfaces, and well-defined compositions. Clays are abundant, low-cost, and environment friendly and thus have been used by humanity for various applications. Historically, there is evidence of the use of clays for medicinal purposes as early as 2500 BC in the Mesopotamian civilization to treat wounds and prevent hemorrhages.^2^ In addition, clay-based materials were used as remedies for several diseases and treatment of wounds and skin afflictions, as reported in documents dating to 1500 BC.^3^ The primary objective in studies of clay materials was, and indeed still is today, the determination of the fundamental factors that control their mechanical and biological properties. To date, clays or silicates and biomedical applications have been addressed in 1090 publications with 31 777 citations based on the ISI Web of Science search on April 13th, 2022. There are also several excellent recent reviews of various types of clays in biomedical applications.^4–15^

The role of silicates and nanoclays, in particular on cellular response, is an important area of research. Previous studies show that nanoclays exhibit an ability to mediate human mesenchymal stem cell differentiation without the use of differentiating media.^16^ Researchers also report using nanoclays to enable osteogenic behavior with human mesenchymal stem cells.^17–21^ Molecular dynamics simulations have probed the interaction between silica particles and integrin molecules- the primary perpetrators of cell adhesion.^19,22^ Experimental studies using next-generation sequencing technology (RNA-seq) have also demonstrated that nanoclays influence over 4000 genes.^23^

## 2. Structure of clays

The mineral structure of clays was first investigated by Linus Pauling using X-ray techniques.^24^ The fundamental components of clay minerals, such as alumina, silica and water, iron, magnesium, alkalis, and alkaline earth, and varying amounts of non-clay-mineral particles like quartz and calcite were also determined.^24^ Clay minerals constitute sedimentary rocks and derived soils made of layered particles that feature one or more phyllosilicate minerals.^25–27^ The phyllosilicate minerals are composed of a silicate crystal structure with various elemental compositions and physical dimensions.^28^ Clay minerals can be of natural and synthetic origin, and their basic building blocks consist of alternating tetrahedral SiO_4_ and octahedral AlO_6_ sheets.^25^ They are categorized into different families by their specific structures and compositions due to the varying ratios of the sheets, such as (a) 1 : 1- has one octahedral layer linked to a tetrahedral one; (b) 2 : 1- has two tetrahedral sheets on either side of an octahedral, and (c) 2 : 1:1- has a positively charged brucite sheet sandwiched between layers that restrict swelling.^29,30^ Moreover, clay minerals can also exist as elongated fibrous structures, which consists of ribbon-like layers of tetrahedral units bound by a central octahedral unit with shared oxygen.^31^Table 1 lists the common clay types according to the structure.

**Table 1:** Common clay types according to the structure

Clay structure		Clay types
Layered	1 : 1	Halloysite, kaolinite, rectorite
	2 : 1	Bentonite, hectorite, LAPONITE®, montmorillonite, sepiolite, saponite, vermiculite, illite, muscovite, biotite
	2 : 1 : 1	Chlorite
Fibrous		Attapulgite

X-ray and electron diffraction techniques helped identify the crystalline structure of the clay minerals along with their atomic structure.^24^ Individual natural clay particles are smaller than 4 μm in diameter, whereas colloidal-clay particles are finer (<1 μm in diameter) and are found as layered silicates^32^ Clay minerals have a general chemical formula of (Ca, Na, H)(Al, Mg, Fe, Zn)_2_(Si, Al)_4_O_10_(OH)_2−*x*_H_2_O, where *x* represents the amount of water.^28^ Environmental changes, such as humidity content in the surrounding, can cause the clay to absorb or lose water, resulting in variable specific gravity of any clay.^28^ Thus, the physical characteristics of clays are essential in defining the various types of clays.

The general structure of clay particles is recognized as layered or fibrous.^24^ Each layer comprises two types of structural sheets: tetrahedral and octahedral. While the former is composed of silicon–oxygen tetrahedra linked to neighboring tetrahedra by sharing three corners, resulting in a hexagonal network, and the remaining fourth corner of each tetrahedron forms a part of the adjacent octahedral sheet, the latter is usually composed of aluminum or magnesium in six-fold coordination with oxygen from the tetrahedral sheet and with hydroxyl.^29^ The two sheets form a layer, and several layers may be joined in a clay crystallite by interlayer cations, van der Waals force, electrostatic force, or hydrogen bonding.^28^ The fundamental structural units are silica tetrahedron and aluminum octahedral. The cation-Si^4+^ is fourfold and possesses tetrahedral coordination with oxygen, while the cation, Al^3+^, occurs in sixfold or octahedral coordination.^28^

Clay minerals have four general structural types: layered structures of three types (1 : 1, 2 : 1, 2 : 1 : 1) and one fibrous structure. The 1 : 1 type comprises unit layers, with each layer consisting of one silica tetrahedral sheet and one alumina octahedral sheet bound together in a common sheet with shared oxygens.^29^ The units are stacked one above the other in the *c*-axis direction. In case of substitutions of cations within the structure, the clay is balanced electrically.^29,30^ Through isomorphous substitution Si^4+^ can be replaced by Al^3+^ in tetrahedral coordination, and replacement of Al^3+^ is possible by Mg^2+^, Fe^2+^, and Fe^3+^ in octahedral coordination.^28^ This, however, mainly results in charge changes. The 2 : 1 type comprises two silica tetrahedral sheets with a central octahedral sheet bound by two common sheets with shared oxygens.^29^ Here, a considerable number of Si^4+^ in tetrahedral positions are replaced by Al^3+^ and the octahedral positions may either be filled (trioctahedral) or two-third filled (dioctahedral) with aluminum, iron, or magnesium, alone or in a combination.^29,30^ The layers are stacked one above the other in the *c*-axis direction.

However, specific clay minerals from the same type vary based on the occupants of the cation positions, charge on the lattice, nature of the balancing interlayer cations, and stacking arrangements.^29^ In fact, the 2 : 1 : 1 type is an octahedral sheet adjacent to a 2 : 1 layer, where a considerable number of silica is replaced by alumina. This substitution is balanced by interlayer magnesium surrounded by hydroxyls in octahedral coordination in a brucite structure.^29^ To further balance such substitutions in the silicate layer, magnesium is partly replaced by aluminum or ferric iron to provide the excess positive charge that's required.^30^ The fibrous type of clay minerals is composed of ribbon-like layers of two tetrahedral sheets held together by a central octahedral sheet through shared oxygens; which results in a gutter-and-channel-type structure.^31^ The dominant component of the octahedral positions is magnesium, balanced electrically with some replacements by aluminum and iron. In this type of structure, it is found that the components of octahedral positions vary greatly, resulting in varied compositions, namely, palygorskite, *para*-montmorillonite, and *para*-sepiolite.^31^ Moreover, this type binds montmorillonite so firmly that it is difficult to isolate a pure form.^29,30^ Therefore, the structure of clay minerals can be explained in terms of the arrangement of tetrahedral and octahedral sheets. Clays have a hierarchical structure starting with individual clay sheets at the basic level, followed by the layered structure that defines the clay type and the layered structure is further stacked vertically to form a tactoid. The tactoids, in turn, are clustered in different orientations to form an aggregate.^33^ In this review, we will discuss the structure, properties, and biomedical applications of a few major clay types of all the mineral groups mentioned in Table 1.

### 2.1 Structure of kaolinite

**Fig. 1:**
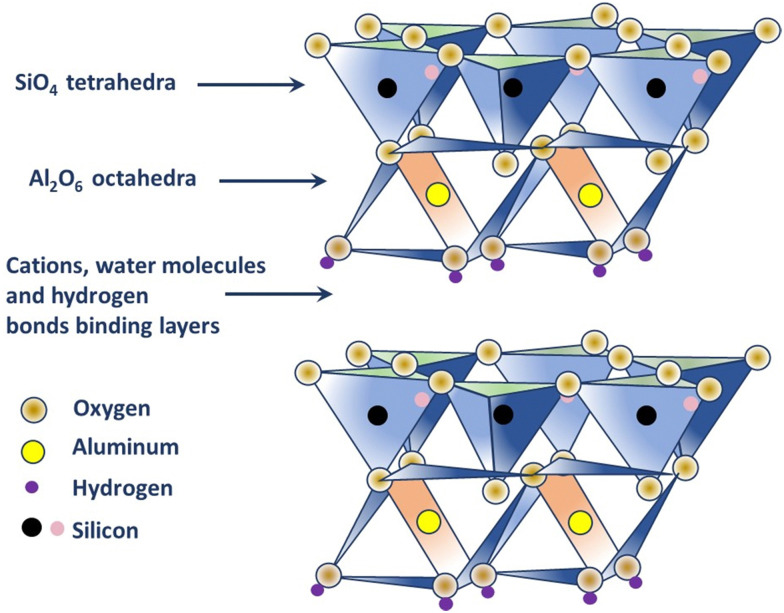
Structure of kaolinite.

Kaolin is a type of clay, also known by the term ‘China clay,’ composed of kaolin group of minerals, namely, kaolinite, halloysite, dickite, and nacrite; where kaolinite is the most common mineral.^25^ Each of the members of the group has the same formula, [Si_4_]Al_4_O_10_(OH)_8_·*n*H_2_O (*n* = 0 or 4), indicating that they are polymorphs, *i.e.* they have the same formula but different structures.^25,26,34^ Kaolinite is white or near-white in color and classified as a two-layer clay (1 : 1 type), where silicate (s) sheets are bonded to the aluminum oxide/hydroxide layers called gibbsite layers through octagonal hydroxyls (refer to Fig. 1).^25,26^ Different cations present, such as K^+^, Ca^2+^, and Mg^2+^ in kaolinite neutralizes the negative charges of the oxide ions. In fact, the structure has a limited substitution of other elements, for example, a few Al substituted by Fe and Si substituted by Al, which results in minimal charge on the kaolinite layer and, subsequently, a low cation exchange capacity (1–15 m equiv. per 100 g).^25^ The hydroxyl groups that occur at the edge of the kaolinite crystal, due to the broken bonds, are considered to be the most reactive sites of the structure (about 10% of the whole surface) and can be negated by the addition of a small amount of chemical dispersant; thus making kaolinite hydrophilic in nature.^25,26,35^ Electron micrographs produced by K. M. Towe in 1961, explained kaolinite as ‘aggregations of book-like particles hexagonal outlines’.^25,26^ A representative structure of kaolinite is shown in Fig. 1.

Due to its relatively low surface area and charge compared to smectite, palygorskite, and sepiolite, kaolinite exhibits low absorption and adsorption.^25^ However, modified forms of kaolinite contribute towards improving specific characteristics. Many commercial products are available for rapid blood clotting abilities that contain kaolinite. Here, the increased surface area in the nanocomposite promoted good absorption capacity and, subsequently, hemostasis.^36^ A similar result was observed in a drug delivery application^37^ where modification of kaolinite with methoxy group increased the interlayer spacing between the sheets, providing a larger surface area for drug loading. Controlled drug release can be influenced by the type of bond formation, charge, and pH. A study showed that Doxorubicin exhibited an increase in drug release rate at pH 5.5, mainly attributed to the decrease in electrostatic interactions between positively charged drug and negatively charged kaolinite surface at low pH.

### 2.2 Structure of halloysite

**Fig. 2:**
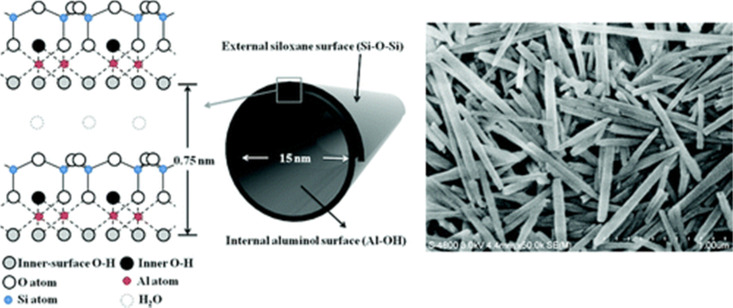
Crystalline structure and FE-SEM image of halloysite nanotubes.^40^

The major source of halloysite is on the North Island of New Zealand.^38^ The general stoichiometry of halloysite is Al_2_Si_2_O_5_(OH)_4_·*n*H_2_O, where *n* = 4 for 1.0 nm wall-packing spacing and *n* = 2 for 0.72 nm (dried sample). It has a similar composition as kaolinite, except that it contains an excess of water molecules between the layers, and successive silicate layers are shifted randomly in both directions (*a*- and *b*-axis).^29,39^ It falls under the 1 : 1 type and exhibits a two-layered tubular structure.^29,30^ These layers may be curled or rolled up, resulting in a structure that is the combination of the geometry of nanotubes with the chemistry of kaolinite.^29^ These exhibit an external diameter of 40–60 nm, an internal diameter of 10–15 nm, and a length of 700–1000 nm. Generally, the external surface of the group has a tetrahedral sheet composed of siloxane groups, whereas the inner surface comprises octahedral sheets of alumina groups. It forms a cylindrical shape due to the mismatch in the alignment of the two layers.^29^ One of the significant advantages of halloysites, with respect to other layered structures, is their weak secondary interaction among the nanotubes because it allows them to disperse easily in a polymer matrix.^39^ Crustal structure of halloysite and an electron micrograph of halloysite tubes is shown in Fig. 2.

### 2.3 Structure of montmorillonite

**Fig. 3:**
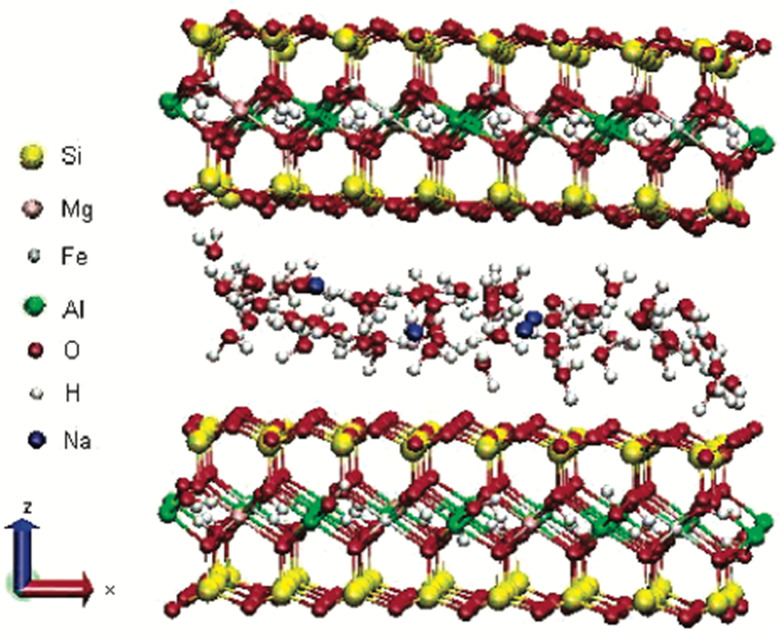
Structure of Na-montmorillonite with two water layers.^45^

Montmorillonite is a layered silicate named after ‘Montmorillon’ in France. It is composed of extremely small units of plate-shaped particles with an average diameter of 1 μm.^29,39^ It is a member of the second structural category, *i.e.*, 2 : 1 layered type, and one of the commonly used minerals from the smectite clay group.^26^ Smectite is the name given to a group of Na, Ca, Mg, Fe, and Li–Al silicates.^25^ There are considerable substitutions in both tetrahedral and octahedral sheets of the structure, which lead to charge imbalances (approximately −0.66 per unit cell).^29^ This charge deficiency is balanced by a variety of interlayer cations that are loosely held and exchangeable.^29^ Layers of water or other polar molecules of variable thickness may enter between the successive silicate layers, separating them^29,41^ with the orientations of silicate tetrahedra oriented with the water molecules.^42^ Thus, if the exchangeable cation is majorly Na, the specific mineral is Na-montmorillonite, and if it is Ca, it is a Ca-montmorillonite.^25,26^ The chemical formula is (Na, Ca)_0.33_ (Al, Mg)_2_ (Si_4_O_10)_(OH)_2_·*n*H_2_O.^39^ As shown in Fig. 3, sodium-montmorillonites generally have one water layer in the interlayer position. While, Ca-montmorillonites generally have two water layers which account for the basal spacing on the X-ray diffraction pattern of 15.4 Å for a Ca-montmorillonite and 12.6 Å for a Na-montmorillonite.^26^ The thickness of the interlayer zone varies with the nature of the interlayer cation and the amount of water or other polar molecules present.^29,43,44^ Montmorillonite has an expanding lattice with a variable *c*-axis dimension and population at the octahedral positions, which may be dioctahedral or trioctahedral.^29^

Montmorillonite is widely accepted for use in polymer nanocomposites because of its easy availability, well-known intercalation or exfoliation chemistry, high surface area, and high surface reactivity.^39,46–48^ Interactions of silicate tetrahedra in montmorillonite with water and other fluids are investigated extensively for geotechnical and environmental applications.^49–52^ These studies present an excellent foundation for the use of silicate structures in biomedical applications.

### 2.4 Structure of LAPONITE®

LAPONITE® is a trioctahedral smectite clay composed of layered synthetic silicates amalgamated from inorganic mineral salts.^53,54^ Since its discovery in 1965,^55^ its been extensively investigated for many applications. This synthetic clay often has a distinct advantage over natural clays because naturally occurring clays can contain impurities that are difficult to separate from the clay.^53^ Thus, LAPONITE® was synthesized from hectorite by controlling chemical formulations, temperatures, and pressures to precisely control their size, shape, and chemical composition.^55,56^

**Fig. 4:**
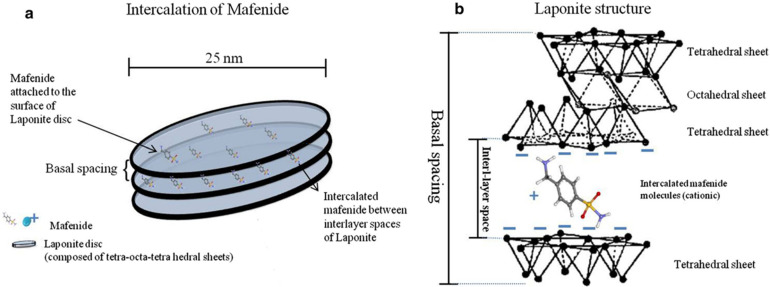
(a) Schematic view of nanosize LAPONITE® discs and inter-layer space between these discs; (b) the chemical structure of LAPONITE® discs and intercalation of cationic ions and drugs (*e.g.*, mafenide) between the inter-layer space.^58^

LAPONITE® is a pure white, free-flowing, non-dusting powder with a bulk density of 1.0 in dry form.^53^ Upon dispersing it in water, it forms a colorless gel with colloidal particles.^53^ Its structural composition consists of an octahedral sheet of magnesium oxide between two parallel tetrahedral sheets of silica, *i.e.*, it belongs to the 2 : 1 smectite group.^39,57^ As compared to montmorillonite, LAPONITE® has a relatively small particle size. Its disc-shaped geometry is characterized by layered hydrous platelet of diameter 20–50 nm and thickness of approximately 1–2 nm (Fig. 4(a)), resulting in a large total surface area and cation exchanging capabilities (Fig. 4(b)).^39^ The empirical formula of this 2D nanoclay is (Na^+^_0.7_[(Si_8_Mg_5.5_Li_0.3_)O_20_(OH)_4_]^−0.7^).^57^ LAPONITE® and montmorillonite have similar structures except for the interstitial charge deficiency created by the replacement of Mg^2+^ with Li^+^.^39^ The cation exchange capacity of LAPONITE® is 0.55 m equiv. per gram.^39^

LAPONITE® nanoclay exhibits dual charge distribution, with a permanent negative charge on the surface of the particle and a positive charge along the edges contributed by its unique composition and size.^53,57^ The hydrophilic properties and large surface area (approximately 345 m^2^ g^−1^) of LAPONITE® enable physical interaction with a wide range of biomolecules.^53^ These properties of LAPONITE® have attributed to its application in therapeutic drug development, regenerative medicine, and additive manufacturing.^39,57^ A study demonstrated a more than two-fold reduction in the clotting time upon adding 2% nano-silicate to 1% kappa-carrageenan hydrogel.^59^ Schmidt and co-workers demonstrated an increase in cell adhesion and a flat and well-spread cell morphology upon increasing the content of LAPONITE® in a nanocomposite film.^60^

## 3. Cellular response of clays

Clays in general and nanoclays, in particular, elicit favorable responses from human cells. Human mesenchymal stem cells are reported to differentiate into osteoblastic lineages on nanoclay scaffolds.^16^ Various researchers have proposed different mechanisms of clay bioactivity, including protein localization, modulation of cell adhesion, biomineralization, and the potential of clay nanoparticles to impact cell differentiation. For example, poly(ethylene) glycol (PEG/PEO), like polymeric hydrogel, is non-fouling, hydrophilic, and does not promote cell or protein adhesion.^61,62^ However, LAPONITE® inclusion in PEG hydrogels at 40–70% (wt%) was demonstrated to improve cell adhesion, proliferation, and spreading of MC3T3-E1 mouse preosteoblasts,^60,63^ NIH 3T3 mouse fibroblasts,^64^ and human bone marrow stromal cells (hBMSCs)^65^ in a clay concentration reliant manner. Likewise, the addition of montmorillonite to polyurethane (PU),^66^ gelatin-cellulose,^67^ and chitosan-based scaffolds^68^ resulted in the clay-dependent favorable effects on cell proliferation and spreading, demonstrating that direct cell-clay interactions promote cell adhesion. Recent studies report that the presence of hydrophilic functional groups (Si–OH and Al–OH) in clay nanoparticles such as those from halloysite nanotubes improve the water absorption to the matrix enhancing surface hydrophilicity, promote cell adhesion and proliferation over the surface of scaffolds.^69,70^ A next probable mechanism is the elevated regional concentrations of divalent cations, like Ca^2+^ or Mg^2+^, which exchange favorably on clay surfaces than monovalent ions due to their higher charge density.^71^ Such divalent cations play essential roles in cellular adherence to biomaterial surfaces, which are regulated primarily by the activation of adhesion proteins of the integrin family.^72,73^ It is reported that the dissolution of LAPONITE® occurs in an aqueous environment resulting in the production of Mg^2+^ ions^74^ which has been shown to promote cell adherence to biomaterial surfaces.^72^

Several studies have also described clay nanoparticles’ capability to improve osteogenic differentiation of mesenchymal stem cells (MSCs) and osteoprogenitor cell populations, even without using standard osteogenic supplements like dexamethasone, β-glycerophosphate, and ascorbate-2-phosphate.^17,21,75^ However, the mechanisms involved in clay-induced osteogenic differentiation are still poorly understood.^76^ According to prior research, clay degradation products may have a crucial role in clay-linked osteogenic bioactivity.^77,78^ In the case of LAPONITE®, nontoxic degradation products, such as Si(OH)_4_, Li^+^, and Mg^2+^, have been associated with enhanced osteogenic cell function. For example, orthosilicic acid stimulates osteoblast differentiation and collagen type 1 synthesis.^79^ Magnesium ions are engaged in initiating osteogenesis-governing pathways (PGC-1α and HIF-1α)^80,81^ and are required for integrin adhesion to biomaterial surfaces.^72^ Lithium is known for initiating canonical Wnt-reactive osteogenic genes *via* GSK3β inhibition.^82^ Clay mineral dissolution generally occurs in aqueous environments. For example, a prior study on silk-MMT clay for bone tissue formation reported the dissolution of clay particles and the presence of silica ions in culture media,^83^ which has been proven to enhance the expression of osteogenic biomarkers.^84,85^ Clays such as halloysite,^86,87^ MMT,^16,83^ and attapulgite,^88^ with various dissolution products have also been shown to have favorable osteogenic effects.

Additionally, several physical and chemical interactions, including electrostatic interactions, cation exchange, hydrogen bonding, hydrophobic affinity, and van der Waals forces, are involved in the adsorption and attachment of protein molecules to clay particles.^89^ Clays can adsorb charged protein molecules due to their surface charge distribution caused by electrostatic interactions.^90^ However, these interactions are also affected by positively and negatively charged states of protein complexes in an adsorption pH environment.^91,92^ In combination with electrostatic forces and cation exchange, the existence of hydrophilic and hydrophobic regions on the clay surface also contributes to protein molecules' interaction with clays.^93^ To maintain structural stability, the adhesion of protein molecules on the hydrophobic areas of clays can lower the free energy system.^94^ However, environmental variables such as pH of the media can also influence protein–clay interaction.^95^

Another mechanism of clay bioactivity is integrin-mediated cell adhesion. Cell adhesion is a fundamental necessity for the survival of anchorage-reliant cells on the matrix surface. The integrin-mediated adhesion of cells to the extracellular matrix tightly regulates the cell development cycle in mammalian cells.^96^ Earlier studies on the activation of integrins and intracellular components by various inorganic materials revealed various responses. For example, consil® bioactive glass particles with comparable degradation products to silicate nanoparticles governed specific cell signaling pathways comprising the ERK and p38 MAPKs, αV integrin, and the immediate early gene c-Jun.^97^ Using calcium silicate cement with varying Si/Ca molar ratios, researchers discovered that Si-rich cement triggered α2β1 integrin expression and p38 and ERK signaling pathway activation very efficiently than Ca-rich cement. However, Ca-rich cement triggered αvβ3 integrin expression.^98^ Lastly, according to an integrated experimental and molecular modeling study on silica-based biomaterial, the binding of αVβ3 integrin to the silica surface stimulates its activation. Which initiates an activation cascade comprising the three MAPK pathways: p38, ERK, and JNK, which further activate Runx2, responsible for the induction of bone extracellular matrix proteins.^99,100^ However, the mechanism by which certain inorganic elements, such as silica, activate the integrins remains unclear, opening new opportunities for understanding and manipulating clay-based nanocomposites to accomplish specific cell responses.

Nanocomposite bioinks are another promising platform for bioprinting the cells in three dimensions, resulting in cell-laden constructions that aim to assist tissue repair and functionality. Bioprinting, also known as 3D printing, is a revolutionary innovation that can generate 3D scaffolds with excellent functional properties and all the biological cues for faster tissue regeneration.^101,102^ The majority of polymers and nanocomposites can be printed efficiently using extrusion-based 3D printing technology.^103^ However, the appropriate viscosity of the bioinks or cell-laden materials is critical, particularly for cell printing. For example, Any hydrogel with a viscosity less than 300 mPa s is unsuitable for maintaining the shape integrity of the desired 3D build.^104^ However, higher viscosities of bioinks are not suitable for cell printing because they need more pressure to flow, and the embedded cells eventually undergo more significant shear stress, which might injure the cells.^105^ Nanoengineered bioinks have created a new avenue for improving the shape of 3D printed scaffolds while exhibiting various exceptional properties such as controlled drug discharge, biomineralization, mechanical strength, quick gelling, self-crosslinking, and conductivity.^106^ Nanoclays were an excellent additive for creating nanoengineered bioinks over various nanomaterials.^107^ Their biocompatibility, water solubility, and significant influence on rheological and mechanical properties have contributed to their prominence in bioink reinforcement.^108^ Nanoclays disperse in water and can improve the flow behavior, shape restoration, and bioactivity of bioink. By adjusting the viscosity and shear thinning characteristics of the pre-gel solution as a function of clay concentration, recent methods have optimized 3D printing bioinks to create robust hydrogels in various complicated forms.^109^ For example, Cell-laden LAPONITE®-based nanocomposite bioinks demonstrated better printing properties that enabled the creation of complicated forms and cell spreading of various encapsulated cells.^110–112^

## 4. Biomedical applications of clays

### 4.1 Hemostatic agents

Trauma accounts for a significant proportion of mortality worldwide. Excessive bleeding is always considered the main reason for traumatic death.^113^ In most cases, trauma-related mortality occurs in the first few hours. Biological processes are triggered to initiate blood coagulation to combat blood loss due to injury. Initially, blood coagulation factor XII converts into an active form FXIIa that triggers the intrinsic pathway of blood coagulation and platelet aggregation. Subsequently, FXIIa promotes FXIa activation that further binds to FIX and FVIII and triggers their activation. Such complex converts FX to FXa, which further binds to FVa to form prothrombinase and leads to the release of thrombin (FIIa). FIIa promptly converts fibrinogen (FI) to fibrin (FIa), thus promoting crosslinked polymerization of fibrin to form blood clots.^114^

In case of deep injuries where biological routes fail to halt the bleeding, external topical hemostats contribute maximally to regulate excessive bleeding. The efficiency of the hemostat is based on its capacity to absorb blood plasma that allows the clotting factors and platelets to concentrate, biocompatibility with blood cells, and activation of the coagulation cascade. LAPONITE®, kaolinite, and MMT nanoclay-based hemostatic agents have been extensively used due to their unique characteristics.^115,116^ LAPONITE® nanoclay contains a dual charged surface, high cationic exchange capacity, and biocompatibility under physiological conditions. It has been reported that incorporating LAPONITE® in hydrogels can improve their hemostatic efficiency. A recent study showed a decrease in clotting time of kappa-carrageenan hydrogel (∼4 min) with an increase in the concentration of LAPONITE® nano-silicates. Blood in contact with pure kappa-carrageenan hydrogels initiates clotting in ∼7 min which is equivalent to the coagulation time of human blood under normal conditions (5–7 minutes). However, adding 2% nano-silicate to 1%, kappa-carrageenan hydrogel reduced the clotting time by more than two folds (<3 min). The possible reason for the decrease in clotting time may be attributed to the reduction in the zeta potential of the hydrogel surface in the presence of nano-silicates, resulting in a highly negatively charged surface of hydrogels. The negatively charged surface activates platelets and triggers the intrinsic coagulation pathway *via* clotting FXII.^59^ Similar effect has been observed in gelatin and LAPONITE®-based hydrogels, where clotting time is reduced by 77% due to a reduction in the zeta potential of the gelatin surface after LAPONITE® addition.^117^

**Fig. 5:**
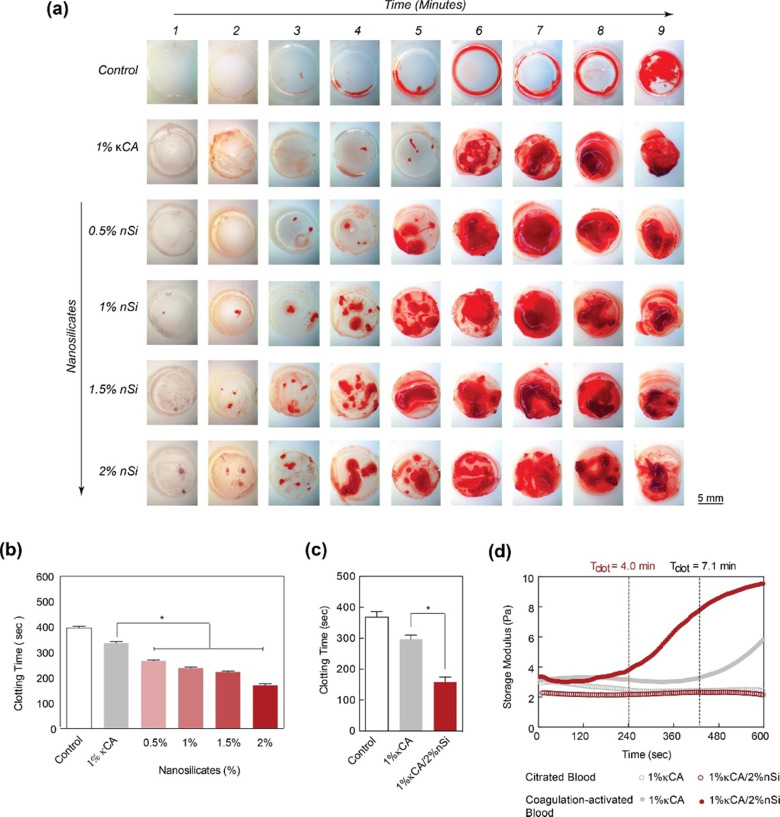
LAPONITE® nano-silicates reduced blood clotting time. (a) The images show clotting blood time with respect to increasing nano silicate concentration in κCA hydrogels, indicating that increasing the concentration of nanosilicates significantly decreases the clotting time. (b) and (c) A quantitative analysis of clotting time *vs.* nanosilicate concentration, representing a decrease in clotting time by more than two-fold with the addition of 2% nanosilicates w.r.t to control. (d) The clotting time on κCA and κCA/Si nanocomposite was also determined by evaluating storage modulus over time, indicating accelerated clotting after adding nanosilicates.^59^

Kaolinite-based hemostats have also gained considerable attention due to their outstanding ability to induce blood clotting and excellent biocompatibility. The ability of nanocomposite hydrogels to obtain hemostasis was studied by measuring blood clotting time (Fig. 5(a) and (b)).^59^ It was observed that the concentration of nanosilicate in natural polysaccharide and κ-carrageenan (κCA) based hydrogel influences the clotting kinetics of whole blood (Fig. 5(c) and (d)).

**Fig. 6:**
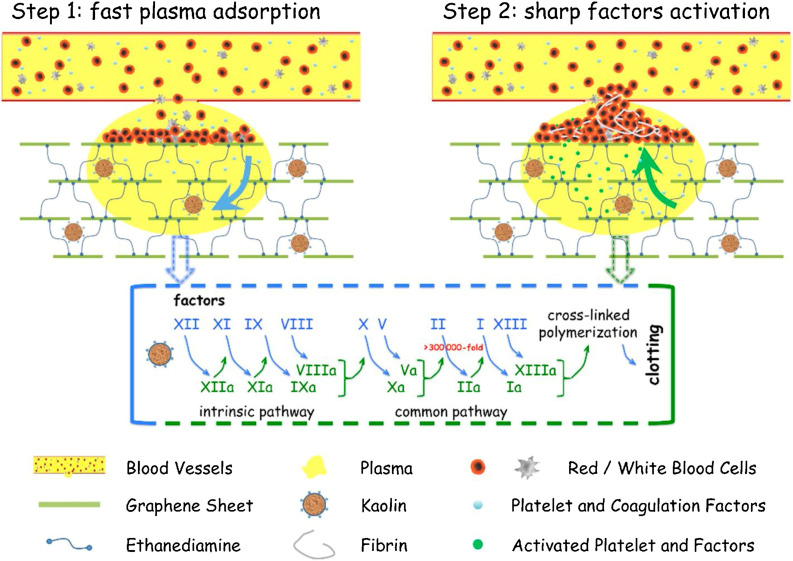
Schematic showing the hemostatic mechanism for the graphene–kaolin composite sponge (GKCS).^123^

Modified forms of kaolinite, such as iron oxide kaolinite nanocomposite, showed better results in terms of blood clot formation than kaolinite alone. A study showed that α-Fe_2_O_3_-kaolinKAc nanocomposites achieved rapid hemostasis due to their efficient water absorption capacity that concentrates blood platelets, RBCs, and clotting factors. In addition, α-Fe_2_O_3_ and kaolinKAc synergistically activated the intrinsic coagulation pathway by stimulating FXII to FXIIa conversion.^118^ In 2013, QCG, a commercial kaolin-based hemostat, was approved by the Food and Drug Administration (FDA) due to its high efficiency in controlling excessive bleeding without any risk of thermal injury. Previously FDA-approved zeolite-based hemostats generated spontaneous exothermic reactions, leading to thermal injury and necrosis of surrounding tissues.^119^ Testing QCG in large animals with severe wounds in the liver^120^ and femoral artery^121,122^ demonstrated that the bleeding stopped within a minute of its application. A graphene–kaolin composite sponge (GKCS) was recently introduced as a hemostat where kaolin and graphene oxide were mixed in different ratios. Among them, the 1 : 1 w/v ratio showed promising results that effectively showed promising results of stopping bleeding in 73 seconds in the rabbit artery injury model. Due to remarkable plasma absorption capacity and overall increased negative potential, GKCS led to rapid activation of blood clotting factors and platelet aggregation, as shown in Fig. 6.^123^

The hemostatic performance of MMT nanoclays is also governed by their ability to swell and charged stimulation of activating blood coagulation. Some studies have evaluated the safety of smectite granules (MMT is the main smectite mineral) containing hemostat, WoundStat™ in a porcine model and revealed that smectite granules caused potential thrombosis upon blood contact.^124,125^ The studies showed that despite adequate debridement, residues of smectite granules remained in the lumen of arteries, eventually causing thrombosis. Another study also showed significant cytotoxicity of montmorillonite on human umbilical vein endothelial cells, causing 100% cell lysis after 24 hours of cell contact.^126^ However, more studies are needed to evaluate the risk of thrombosis.

**Fig. 7:**
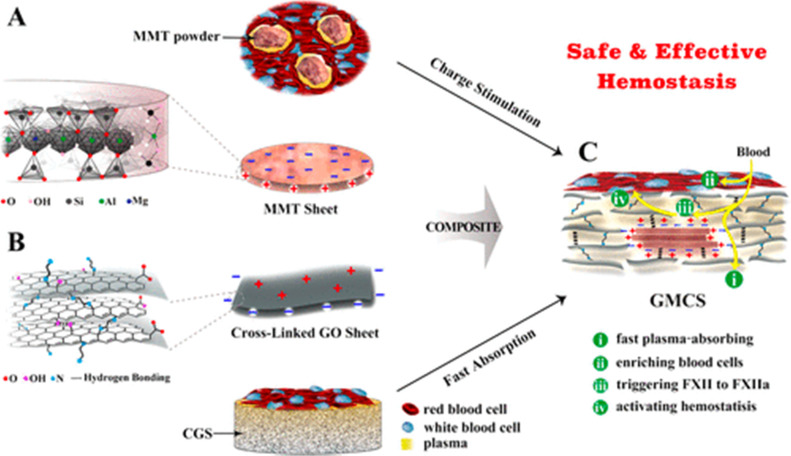
Schematic shows MMT and graphene oxide's synergistic effect for accelerating hemostasis in the graphene-MMT composite sponge (GMCS). (A) The MMT sheets possess a negative charge on their surface and a positive charge at their edges (B). The crosslinked graphene sheets possess a positive charge on their surface and a negative charge at the edges. (C) The crosslinked graphene sponge (CGS) accelerates hemostasis by rapidly absorbing plasma and enriching blood cells on the sponge surface, while MMT activates the clotting factor.^127^

To eliminate these side effects, Li and Co-workers have developed a graphene-MMT composite sponge (GMCS) that prevents direct interaction between MMT and blood and rapidly stops bleeding in 85 seconds in the rabbit artery injury model. Due to strong interactions between MMT and graphene oxide, MMT is embedded tightly within graphene sheets, preventing its leakage from GMCS (Fig. 7).^127^

Halloysite nanotubes (HNTs) are known to promote blood coagulation and are non-hemolytic in nature. A study demonstrated that HNTs showed less than 0.5% hemolytic ratios when interacted with rabbit blood.^128^ There are, however, few scientific reports available on hemostatic or wound healing applications. Its application as a wound healing composite has been investigated well. A study showed that with the addition of HNTs in chitosan-HNTs composite sponges, the compression strength of composite sponges was increased about 8.8-fold along with an increase in clotting ability to 89.0% compared to pure chitosan sponges. The increased clotting percentage of composites was directly correlated with increased nano-roughness of the pore-wall of sponges by HNTs that favored entrapment of proteins and increased surface area for cell adhesion.^129^ Recently, cellulose-halloysite hemostatic nanocomposite fibers (CHNFs) were fabricated that showed a faster average clotting time for CHNFs, 67 ± 5 seconds, than the commercial kaolin-based QCG that clots blood in 85 ± 5 seconds. The improvement in the hemostatic ability of CHNFs may be attributed to high clay loading by cellulose fibers and is seven times higher than QCG. In addition, it is reported that neat HNTs coagulate human plasma approximately 1.6 times faster than neat kaolin clays.^130^

### 4.2 Drug delivery

Nanoclays have been extensively studied for their drug and gene delivery applications. Due to the high cation exchange capacity of MMT nanoclays, they have been explored well for targeting and controlling the release of drug molecules. Low adsorption and poor cation exchange capacity of kaolinite limited their application in drug delivery in unmodified form. Thus, modified forms of kaolinite have attained great attention for drug delivery applications. Halloysite nanotubes (HNTs) are often considered a first-choice carrier for drugs among different nanoclays. Its unique tubular structure allows them to load drugs with high capacity *via* adsorption or intercalation; however, the non-degradable nature of HNTs limits its clinical application.

While halloysite nanotubes exhibit a positive charge inside the lumen, which is particularly important for the high loading of anionic drug molecules and negatively charged DNA and proteins into the lumen, the outer surface of halloysite nanotubes is negatively charged, providing a platform for cationic drug adsorption *via* electrostatic interactions. Due to the ease of tailoring inner and outer surfaces with functional groups, HNT offers an efficient system for high drug loading and controlled drug release.^131,132^ Price *et al.* presented a pioneering study in the use of halloysite nanotubes as a drug carriers, proposing loading the lumen of HNTs with saturated drug solutions and followed by their subsequent release.^133^ Thus HNTs have been used for the capture and eventual release of three different compounds: oxytetracycline HCl (a water-soluble antibiotic), nicotinamide adenine dinucleotide (NAD) (a co-enzyme that is essential in several biochemical activities) and khellin.^133,134^

**Fig. 8:**
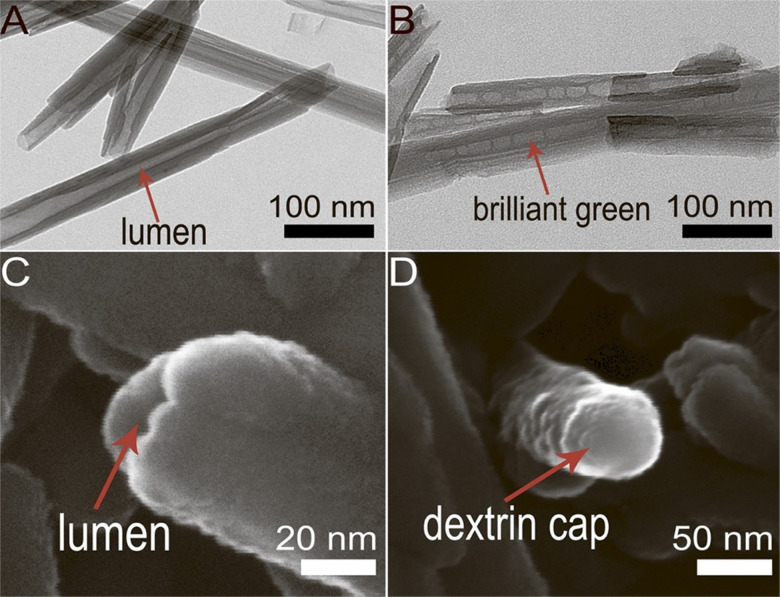
Selective drug delivery by lumen-capped halloysite nanotubes (HNTs) (A) TEM image of HNTs without end-capping; (B) TEM image of brilliant green loaded HNTs; (C) SEM image of HNTs with open lumen; (D) SEM image of HNTs with dextrin capped on the lumen end.^137^

HNT functionalization by grafting silane coupling agents such as 3-Aminopropyltriethoxysilane (APTES) or 3-Glycidoxypropyltrimethoxysilane (GPTMS) is majorly used to modify HNTs for potential drug delivery systems.^7^ A study reported that silane-modified HNTs with organosilane -APTES or GPTMS displayed a much higher drug loading capacity than unmodified HNTs.^135^ Recently, APTES modified HNTs have also been reported as a delivery agent for an antisense gene, oligodeoxynucleotides (ASODNs), targeting the survivin protein to regulate tumor growth.^136^ Controlled and targeted drug release is also attained by other techniques, such as tubular encapsulation and controlled pore openings of the HNT lumen. A study showed selective release of triazole dye brilliant green, loaded inside the lumen of HNTs tube capable of suppressing mitochondria in the malignant cells. The lumen ends were capped with dextrin stoppers *via* vacuum-facilitated deposition that was supposed to seal the drug inside the nanotubes before their internalization. Fig. 8 presents SEM and TEM images of HNTs with and without end-capping. After their internalization, the dextrin coating was hydrolyzed by intercellular glycosyl hydrolases enzyme present inside the lung carcinoma cells, which resulted in the release of brilliant green inside cancer cells.^137^ Another study showed controlled release of brilliant green using a tube encapsulation approach where HNTs were coated with a porous benzotriazole-copper film that controlled the drug release for 10–200 hours. The benzotriazole-copper coating covered the entire tube surface, including tube ends that allowed the slow release of brilliant green from the tube lumen.^138^

Neurological conditions such as epilepsy are found in individuals of all ages, and many antiepileptic drugs have a limited ability to cross the brain–blood barrier.^139^ In a recent work, Lvov and co-workers employed HNTs as drug transporters to cross the brain microvascular endothelial barriers and prolong incremental payload release.^140,141^ HNTs can significantly improve the efficiency of bioactive molecules that have low solubility in water. For instance, HNTs were also successfully loaded with resveratrol, a drug with limited water solubility known for antineoplastic and antioxidant properties.^142^ The trapping of the compounds like khellin in HNTs enabled their long-term release and enhanced the therapeutic profile.^133^

The loading capacity of HNTs can also be enhanced by the acid etching approach,^143^ where alumina content inside the lumen is gradually decreased with acid treatment, resulting in the formation of HNTs with different inner diameters ranging from 15 nm to 46 nm. In contrast, the outer diameter of the tube remains constant. With an increase in the inner diameter of HNTs, the zeta potential of the surface first increases and then decreases, which may affect the drug loading capability of anion drugs inside the lumen.^144^ Enzymes are also intriguing therapeutics with a high intracellular delivery capability. The utilization of a nanocarrier for enzyme delivery allows their protection from proteases.^145^ Further, a prior study demonstrates that enzyme encapsulation using HNTs offers their stabilization at severe temperature and pH levels.^132^

**Fig. 9:**
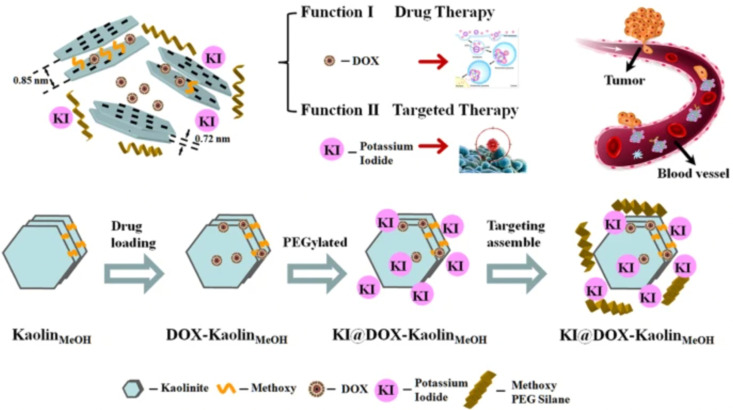
Schematic representation of the KI@DOX-Kaolin_MeOH_ synthesis and Doxorubicin loading for controlled drug release.^37^

Modifying kaolinite with methoxy groups improved its drug loading capacity and release rate. Intercalation of methoxy groups increases the interlayer distance between kaolinite nanoclay sheets from d_001_ 0.72 nm to *d*_001_ 0.85 nm, which provides a relatively large space for drug loading. A study has shown almost twice the loading capacity (20.8 mass%) of methoxy modified kaolinite with an herbicide amitrole (3-amino-1,2,4-triazole) compared to unmodified kaolinite (10.3 mass%) due to an increase in *d*-spacing between nanosheets after modification, resulting in strong electrostatic interaction between intercalated amitrole and the methoxy-modified kaolinite.^146^ However, some drugs do not exhibit strong electrostatic interactions within the layers. These, thus, majorly interact with the external surfaces of kaolinite *via* hydrogen bonding and/or van der Waals forces.^147,148^ An anti-cancer drug, 5-Florouracil (5-FU), showed high drug loading onto the external surface of methoxy-modified kaolinite (40.8 mass%) compared to interlayer loading (14.6 mass%) due to limited interlayer space of the methoxy-modified kaolinite, that was not enough for the crystallization of 5-FU; thus the intercalated 5FU loading capacity was low in their amorphous state.^149^ Controlled drug release is also influenced by the electrostatic interactions between positively charged drug molecules and negatively charged kaolinite surface that varies at different pH. A study showed that Doxorubicin exhibited an increase in drug release rate at pH 5.5, mainly attributed to the decreased electrostatic interactions between positively charged doxorubicin (DOX) drug and negatively charged kaolinite surface at low pH. Fig. 9 displays a schematic representation of the method for synthesizing KI@DOX-Kaolin_MeOH_ and its associated roles in tumor therapy. Generally, cancer cells exhibit a more acidic microenvironment compared to normal cells. Thus, under physiological conditions where pH is 7.4, the release rate of Doxorubicin was low, with a cumulative release of 9.5% over 30 hours. However, at pH 5.5, which is nearly equivalent to the tumor acidic microenvironment, the release rate of the drug was faster, with a cumulative release of 32.5% over 30 hours.^37^

**Fig. 10:**
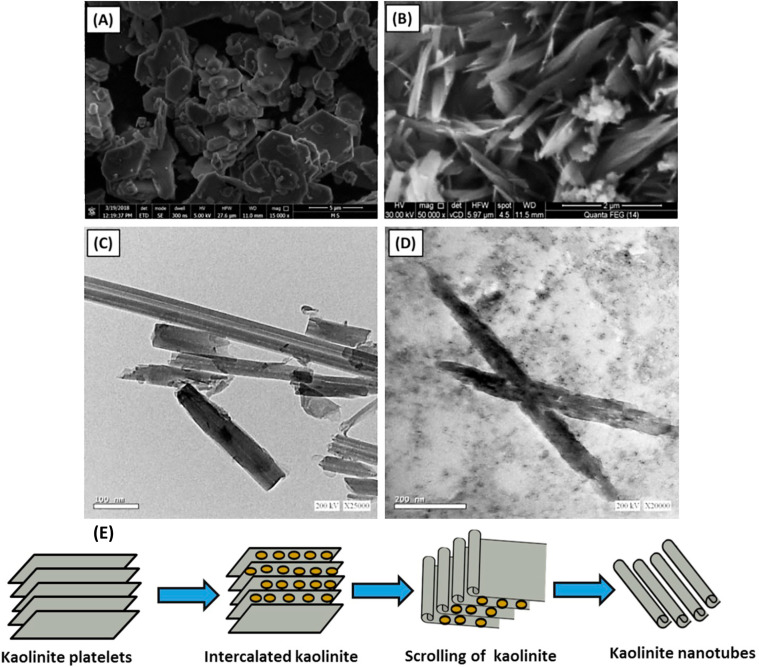
Kaolinite nanotubes for slow drug release (A) SEM of raw kaolinite (B) SEM image of kaolinite nanotubes (C) TEM of unloaded kaolinite nanotubes, and (D)TEM of kaolinite nanotubes loaded by 5-FU drug.^151^

Recently, kaolinite nanosheets have been modified to nanotube structures, showing promising results in high loading capacity and slower drug release rate (Fig. 10).^150^ The nanotubes have lengths ranging from 50 nm to 600 nm, and the internal diameter ranges from 2 nm to 20 nm (Fig. 10(c)). Methoxy-modified kaolinite nanosheets exhibited a relatively fast drug release rate, and it has been reported that 5-FU released almost 100% in only 12 hours. On the contrary, kaolinite nanotubes, encapsulating the same amount of 5-FU drug, released it at a slower rate that continues up to 60 hours. This difference in release profile can be explained by weak hydrogen bonding between adsorbed 5-FU drug molecules and the external surface of kaolinite nanosheets, whereas 5-FU exhibited more affinity within the internal channel kaolinite nanotubes, resulting in controlled release of 5-FU.^151^

LAPONITE® nanodiscs exhibit a similar phenomenon of pH-dependent drug loading and release behavior. A study showed that at pH 3 (acidic condition), the surface charge of edges of LAPONITE® nanodiscs becomes more positive, resulting in strong electrostatic interaction between negatively charged Dexamethasone drug and LAPONITE® nanodiscs. However, at neutral or basic pH, a charge of the face and edges of LAPONITE® nanodiscs remains negative, thus interacting with dexamethasone by physical adsorption.^152^ Thus, alteration in pH could affect the drug loading efficiency of anionic drugs based on the surface charge of LAPONITE® nanodiscs.

### 4.3 Tissue engineering

Tissue engineering is a relatively new field first introduced by Langer and Vacanti^153^ that uses science and engineering principles to reach new frontiers in regenerative medicine through the use of biodegradable porous structures called scaffolds seeded with human cells to enable the development of new tissue while scaffolds degrade. Tissue engineering helps improve, maintain, and/or restore tissue functions in the human body. Nanoclays have been incorporated into polymers due to significant improvement in mechanical and thermal properties of the polymers.^154^ The incorporation of nanoclays into polymers requires the use of modifier molecules. The mechanisms of property improvement due to nanoclays are described by the Altered Phase theory, wherein a significant portion of the polymer is influenced by interactions with clay particles.^48^ Many efforts have gone into developing polymer-nanoclay composites to enable tissue-engineered tissues, particularly bone.^155^ The altered phase theory also allows a way to develop engineered nanoclays with specific modifications to elicit improved properties.^156^ These composites additionally also provide enhanced cell proliferation and adhesion.^157^ Also, based on the desired applications, nanoclay fillers are added to improve bond strength, tailor mechanical properties, affect *in vitro* degradation rates, and further enhance cell growth. In recent years, it has been found that LAPONITE®, HNTs, and MMT nanoclays have been used for numerous soft tissue and hard tissue engineering applications. Engineered nanoclays modified with amino acids promote osteogenesis without osteogenic differentiation media, indicating a direct interaction between nanoclays and proteins involved in osteogenic pathways. Several studies suggest the role of silicate ions of nanoclays in enhancing bone mineralization by influencing nucleation and deposition of calcium and phosphate inorganic ions into extracellular matrix.

**Fig. 11:**
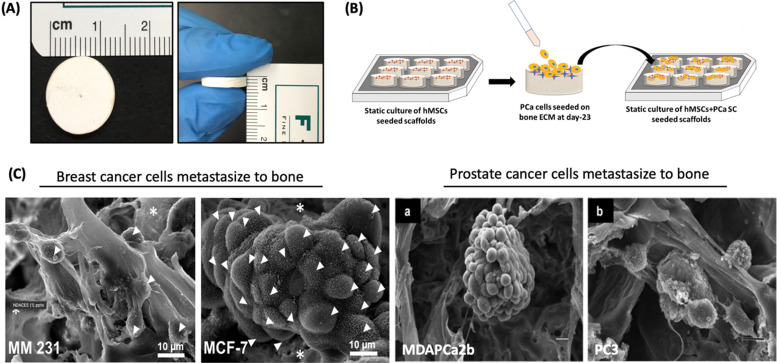
Prostate and breast cancer bone metastasis on bone-mimetic scaffolds (A) Scaffolds dimensions 12 mm diameter and 3 mm thickness (B) Schematic representation of cell seeding on scaffold surface (C) tumor formation on bone microenvironment by breast cancer cells (MM231 and MCF-7) and prostate cancer (MDAPCa2b and PC3).^162–164^

Fibrous polycaprolactone/HNT composite scaffolds have been fabricated for bone tissue engineering by electrospinning.^86^ These scaffolds demonstrated greater protein absorption, enhanced mineralization, and faster proliferation of MSCs seeded on the scaffolds. In a recent study, the synergetic effect between MMT and hydroxyapatite (HAp) was determined for swelling ratio, density, biodegradation, mechanical behavior, decreased degradation, and increased biomineralization.^158^ It was found that the incorporation of MMT was largely responsible for controlling these properties. Kaplan and co-workers studied silk/MMT clay films as a composite with human mesenchymal stem cells (hMSCs) in an osteogenic culture medium.^83^ The results suggested that the composite supported the attachment, proliferation, and osteogenic differentiation of hMSCs, maintaining high cell viability. In a similar approach, the Katti research group proposed using a 5-aminovaleric acid-modified Na-MMT scaffold system for bone tissue engineering applications. Na-MMT nanoclay improved the mechanical properties of polycaprolactone-hydroxyapatite (PCL-HAp) based scaffolds and enhanced the biomineralization of HAp, which is necessary for enhanced bone growth.^159–161^ These scaffolds showed osteogenic differentiation of hMSCs into bone cells without the use of osteogenic supplement.^75^ In recent years, these scaffolds have been used for a novel application of creating the bone metastatic site for prostate,^162–164^ and breast cancer (Fig. 11).^165^ The results showed mesenchymal to the epithelial transition of breast and prostate cancer at the tissue-engineered bone, mimicking realistic behavior of cancer metastasis to bone behavior.

HNTs have also been evaluated for their bone-tissue engineering applications. In one study, HNT-incorporated hydrogels were synthesized by photopolymerizing HNTs and gelatine methacrylate to improve bone regeneration rates.^166^ The incorporation of 7% w/w concentrations of HNTs in hydrogels showed a remarkable increase in compressive modulus up to 0.4 MPa that ultimately improved the mechanical performance of hydrogels. Moreover, HNTs showed enhanced osteogenic differentiation of human dental pulp stem cells (hDPSCs) cultured on these hydrogels due to increased expression of osteogenesis-related genes *in vitro* and *in vivo* conditions. It is also evident from some studies that the internalization of HNT by the cells may have a direct influence on improved osteogenesis. Several other studies also suggest increased bone mineralization by silicate ions of HNTs.^167^

**Fig. 12:**
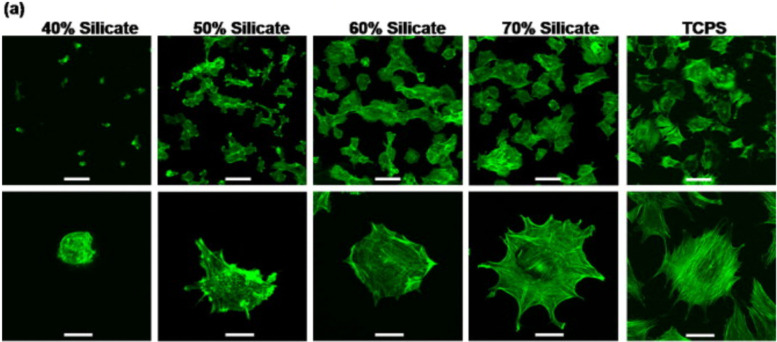
Silicate ions of LAPONITE® enhances cell adhesion and spreading on poly(ethylene)glycol (PEO)-LAPONITE® film surfaces. Preosteoblast cells seeded on LAPONITE® crosslinked PEO nanocomposite films showed better cell spreading and cell adhesion with increasing silicate concentrations, determined by F-actin staining. Scale bar: 100 (top row) and 40 μm (bottom row).^60^

Nanosized LAPONITE® particles can adhere directly to the cell surface^168,169^ or internalize into the cells,^77,78^ inducing osteogenic differentiation of mesenchymal stem cells. These synthetic silicates are dispersed into the aqueous solution and release ions such as sodium ions (Na^+^), orthosilicic acid (Si(OH)_4_), magnesium ions (Mg^2+^), and lithium ions (Li^+^).^170^ These products also play a significant role in cell adhesion. While magnesium ions promote cell adhesion to the substrate by interacting with the adhesion protein of the integrin family, orthosilicic acid and lithium ions are known to promote collagen type I synthesis and Runt-related transcription factor-2 (RUNX2) activity, respectively, thus enhancing osteogenesis.^77^ A recent work shows the role of silicate ions of LAPONITE® in improved cell adhesion, cell spreading, and the osteogenic response of preosteoblasts on LAPONITE® crosslinked poly(ethylene)glycol films to an increase in LAPONITE® content from 40% to 70%. The nanocomposite films containing 70% LAPONITE® content showed a four-fold increase in cell adhesion and displayed a flat and well-spread morphology (Fig. 12). In addition, an increase in alkaline phosphatase activity (by ten-fold) and mineralization was observed on Day 28.^60^ Similarly, increased osteogenic differentiation of rat bone marrow-derived mesenchymal stem cells was observed with the addition of 5 and 10 wt% LAPONITE® nanoparticles in carboxymethyl chitosan (CMC) gelatin-based biocomposite scaffolds.^171^ The self-assembling LAPONITE® gels by Dawson's research group demonstrated the concept of creating regenerative microenvironments using LAPONITE®.^172^ The LAPONITE® gels with different morphologies, like droplets, rings, long-strings, and clay microcapsules within larger clay capsules, were able to flow through syringe needles, re-establish the gel network, and bridge the tissue gaps of approximately 1 cm. Human bone marrow stromal cells encapsulated within these gels and cultured in a chondrogenic inducing medium were able to differentiate towards the chondrogenic lineage. Co-encapsulation of these cells with fibronectin, an adhesion molecule, increased the matrix synthesis and the number of cells expressing Sox-9 transcriptional activator required for chondrogenesis. This group has also prepared clay microcapsules containing different biomolecules which were later immobilised together to form larger clay capsules.

Kaolinite has not been explored in tissue engineering applications. However, few studies suggest the role of kaolin in improving the mechanical properties of the scaffold with better cell proliferation and cell attachment when incorporated as nanocomposites. A study showed an increase in mechanical strength of mesoporous bio-glass scaffolds from 2.6 to 6.0 MPa with increasing concentration of kaolin from 5–20%, while *in vitro* studies showed osteogenic differentiation of rat bone marrow cells.^173^

## 5. Summary and future perspectives

While applications of clays and nanoclays continue to expand in wound dressing, regenerative medicine, and drug delivery, new areas for the use of clays in biomedical applications are indeed emerging. Several emerging areas include clays in dental orthopedics and tissue-engineered therapies for cancer. Bone substitutes are increasingly finding use in the development of metastasis models, and clays are shown to have a powerful role in inducing osteogenic behaviors.^174^ The bone substitutes market globally was valued at $2.9B in 2021, and it is expected to increase to $4.3B by 2028.^175^ Market trends predict a fast-growing need for dental and orthopedic products in the near future.^175^ Likewise, the global hemostasis products market size is expected to rise from an estimated $5.35 billion in 2018 at a CAGR of 8.7% from 2019 to 2026.^176^ The use of clays is an integral component of these products. Nanoclays also participate in a large share of the drug delivery market. In addition, numerous fundamental studies on interactions of biomolecules pertaining to cellular adhesion, proliferation, and mechanical characteristics are underway. Several promising opportunities for manipulating clay-based nanocomposites to accomplish specific cell responses are presented with ongoing experimental and modeling studies on clay–integrin interactions. Novel silicate modifications can be attempted to elicit favorable cellular responses. Novel uses of nanoclays in bioprinting technologies show much promise. Thus, nanoclays present novel capabilities towards physical and biological responses and present the advent of new promising areas.

## Author contributions

Author KSK is responsible for conceptualization, data curation, formal analysis, funding acquisition, project administration, resources, supervision, writing – original draft and writing – review & editing. Author DRK is responsible for conceptualization, data curation, formal analysis, funding acquisition, project administration, resources, supervision, and writing – review & editing. Authors SVJ, HJ and SM are primarily responsible for writing – original draft and writing – review & editing.

## Conflicts of interest

There are no conflicts to declare.

## Supplementary Material
